# Factors associated with prelacteal feeding practices in Debre Berhan district, North Shoa, Central Ethiopia: a cross-sectional, community-based study

**DOI:** 10.1186/s40795-019-0277-8

**Published:** 2019-02-15

**Authors:** Mesele Damte Argaw, Maeza Mitiku Asfaw, Mekonen Birhane Ayalew, Binyam Fekadu Desta, Thandisizwe Redford Mavundla, Kassa Daka Gidebo, Aynalem Hailemichael Frew, Aychiluhim Damtew Mitiku, Alebel Yaregal Desale

**Affiliations:** 1 USAID Transform: Primary Health Care, JSI Research & Training Institute, Inc., P.O. Box 1392 code, 1110 Addis Ababa, Ethiopia; 2 Department of Health Studies, University of South Africa, Pretoria, South Africa; 3 Lalibela Town Health Office, Lalibela, Ethiopia; 4 Woldia University, Woldia, Ethiopia; 5 School of Public Health, College of Health Sciences and Medicine, Wolaita Sodo University, Wolaita Sodo, Ethiopia; 6 USAID Transform: Primary Health Care, Pathfinder International, Addis Ababa, Ethiopia; 7 Clinton Health Access Initiative, Addis Ababa, Ethiopia

**Keywords:** Breastfeeding, Prelacteal feeding, Factors, Newborns, Mother-infant dyads, Ethiopia

## Abstract

**Background:**

Prelacteal feeding is one of the major harmful newborn feeding practices and is top on the list of global public health concerns. The practice deprives newborns of valuable nutrients and protection of colostrum and exposes them to preventable morbidity and mortality. Studying the prevalence and factors influencing the prelacteal feeding practice of mothers will help program managers and implementers to properly address broad major public health problems. Therefore, this study aims to investigate the prevalence of prelacteal feeding practices and its associated factors among mother-infant dyads in the Debre Berhan district of North Shoa administrative zone, central Ethiopia.

**Methods:**

A community-based cross-sectional study design was conducted from January through to April 2014 among 634 mother-infant dyads. The data were entered into EPI Info version 3.5.1. (CDC, Atlanta, Georgia). All statistical analysis was conducted using Statistical Package for Social Sciences (SPSS) research IBM version 20.0. The prevalence of prelacteal feeding was determined using the ‘recall since birth’ method. Multi-variable logistic regression analysis was employed to control confounders in determining the association between prelacteal feeding practices and selected independent variables. Adjusted Odds Ratio (AOR), with 95% Confidence Interval (CI) and *P* < 0.05 was used to claim statistical significance.

**Results:**

The prevalence of prelacteal feeding practice was 14.2% (95% CI: 11.00–17.00%). Slightly greater than half, 48 (53.3%) of prelacteal fed newborns were given butter. Home delivery was a major risk factor for practicing prelacteal feeding. Mothers who delivered their indexed infant at home practiced prelacteal feeding over four folds more than mothers who delivered in a health institution (Adjusted Odds Ratio (AOR) 4.70; 95% CI: 2.56–8.60, *p*-value = 0.001). Mothers who did not initiate breastfeeding within an hour were six times more likely to practice prelacteal feeding (AOR 5.58; 3.21–9.46, *p*-value = 0.001). Similarly, with regards to the occupation of mothers, farmers practiced prelacteal feedings (AOR 4.33; 95% CI: 1.73–10.81, p-value = 0.002) up to four folds more than their counterpart housewives. Mothers who can read and write are 54% less likely to practice prelacteal feeding than their counterpart, illiterate mothers, with (AOR 0.46; 95% CI: 0.22–0.98, p-value = 0.044).

**Conclusions:**

In the Debre Berhan town of North Shoa administrative zone, central Ethiopia, almost one-sixth of mothers practiced prelacteal feeding. Therefore, improving access to information about appropriate newborn feeding practices, encouraging mothers to deliver their babies in health institutions and inspiring them to initiate breastfeeding within an hour of birth is recommended.

## Introduction

During the last two to three decades, more specifically during the life of the Millennium Development Goals (MDGs), from 1990 to 2015, there have been advancements made globally in improving child survival rates. In 2013, the world witnessed the decline of under-five deaths by more than half from the baseline figure i.e. from 12.7 million to 6.3 million [1], 2 years ahead of the set deadline. To ensure the sustainability of these gains, 180 countries have pledged to further reduce maternal, neonatal and child deaths [1]. Moreover, there will be more effort in the coming years to enhance these gains as part of achieving the Sustainable Development Goals (SDGs) set to be achieved by 2030, as infant feeding practices are a component of the second sustainable goal, ‘zero hunger’; and the third sustainable goal of ‘good health and wellbeing for people’. In addition, the effect of breastfeeding on intelligence which includes human capital is relevant to the fourth goal, ‘quality education’, the first goal, ‘no poverty’, and the eighth goal ‘decent work and economic growth’. Finally, by helping to bridge the gap between rich and poor, breastfeeding can contribute to the tenth sustainable goal of ‘reducing inequalities’ [2].

Breast milk is the ideal nourishment for babies during the first 6 months of life. It contains all the necessary nutrients, antibodies, and hormones that a baby needs to thrive. Therefore, the World Health Organization (WHO) recommends early initiation of breastfeeding for newborns preferably within 1 h of birth [3]. Smith et al. (2017) attest that early initiation of breastfeeding protects newborns from infections and reduces newborn mortality [4]. However, recent systematic reviews have documented that globally, only about half of women who give birth begin breastfeeding within the first hour of life [5]. In addition, studies show that in many countries the harmful feeding practice of prelacteal feeding, is often a norm.

According to Mukuria et al. (2006), prelacteal feeding is defined as “giving liquids or foods other than breast milk prior to the establishment of regular breastfeeding [6].” Newborn feeding practices, specifically among harmful traditional practices, prelacteal feeding practices assume top position on the list of global public health concerns. In Ethiopia, among the common prelacteal feeds given to newborns are;, raw butter, sugar infused with water, plain water and diluted milk other than breast milk [7, 8]. Hence, this practice deprives a newborn child of valuable nutrients and protection of colostrum and exposes a newborn infant to preventable morbidity and mortality [6, 9–11].

This harmful prelacteal feeding practice has been widely documented in almost all administrative regions of Ethiopia. The magnitude of prelacteal feeding practices reported in the whole country ranges 15 to 45.5% [7, 8, 10–14]. The predictors of prelacteal feeding practices were found to be: socio-demographic characteristics, Ante-Natal Care, place of delivery, Post-Natal Services and counseling services received on infant feeding and early initiation of breastfeeding [7, 8, 10–13].

During the last two decades, the Federal Ministry of Health developed and implemented the Infant and Young Child Feeding (IYCF) strategy [15]. One of the main interventions implemented to improve the health of a child is to promote exclusive breastfeeding practices for the first 6 months of life [16, 17]. The national flagship community health program, which is implemented through health extension workers, promotes health through engaging households in taking ownership of their own health issues [18]. In addition, since 2012, community health services have been enhanced through the participatory engagement of a women’s group, the, health development army (HDA). The group is responsible for disseminating health information and facilitating the uptake of basic health services [19]. Furthermore, the Health Sector Transformation Plan II (HSTP II 2015/16–2019/20) has set a goal to raise the amount of exclusive breastfeeding practices to 72% by the year 2019 [17]. To achieve this ambitious goal, households will be encouraged and monitored to take up tailored and predefined health promotion, disease prevention, and basic curative interventions. However, considering diversified socio-economic characteristics of the Ethiopian people and the result observed due to the implemented interventions local evidence on prelacteal feeding practices is scarce.

It is important to study newborn feeding practices as a component of optimum breastfeeding practices. Therefore, this study was conducted to assess prelacteal feeding practices and associated factors among mothers of children aged less than 12 months in the Debre Berhan district of North Shoa administrative zone, central Ethiopia. Studying the magnitude and factors influencing prelacteal feeding practices of mothers will help health care providers, child health program managers and implementers to design tailored interventions to reduce prelacteal feeding and promote exclusive breastfeeding practices.

## Methods and materials

### Study design and setting

This community-based cross-sectional study was conducted among mother-infant dyads from March to April 2014, in the Debre Berhan district of North Shoa administrative zone within the Amhara region. The study area is located 120 km away from Addis Ababa, the capital city of Ethiopia. The study area has an estimated total population of 84,920 people [20]. The study was conducted in four of nine *kebeles* the smallest administrative units where 5000 people live per kebele. The data were part of the study conducted to determine factors associated with exclusive breastfeeding practices in Debre Berhan district, central Ethiopia [21].

### Sample size and sampling

Data were collected from 634 mother-infant dyads. The sample size was determined using Cochran’s formula [22] with its presented assumptions:

$$ ni\kern0.5em =\kern0.5em \frac{Z^2\raisebox{1ex}{$a$}\!\left/ \!\raisebox{-1ex}{$2$}\right.p\left(1-p\right)}{d^2} $$

Where ni is the sample size, Z is standard normal variable at 95% confidence level (1.96), P is (0.50) the proportion of mothers who practiced prelacteal feeding in the study area was not known, d is the marginal error (0.05), design effect (1.5) and contingency for non-response (0.10). Therefore, ni = 576, considering 10% for refusal and incomplete data (*n* = 576+ 58 = 634). Based on the assumption of homogeneity of the population, four (2 urban and 2 rural) out of nine kebeles were selected using lottery methods. Following that, a population census was conducted in these kebeles which was used to identify 1177 mother-infant dyads [20]. After developing the sampling frame, study subjects were selected using every other household with systematic random sampling techniques. During data collection, 53 mothers and their index infants were replaced due to incomplete responses and absence of basic information. Mother-infant dyads who lived for more than 6 months in the study area were chosen for the study.

### Data collection tool and procedure

Structured and semi-structured questionnaires were adopted from the Ethiopian Demographic and Health Survey (EDHS) [12] and WHO recommended national assessment tools for infant and young child feeding surveys [23] were applied for this study. The questionnaire was first prepared in English, translated into Amharic, and then back into English to check its consistency using fluent speakers of both languages. The final Amharic version of the questionnaire was used to collect the data. Data on socio-demographic characteristics, maternal health service uptake and infant feeding practices were collected using the ‘recall since birth’ method. The most significant study question asked was, “Before initiation of breastfeeding, was <name of indexed infant> given anything to drink and/or eat other than breast milk?”. The data was then collected through face to face interviews conducted at the study participants’ home. Respondents who were unavailable or absent were revisited.

### Study variables

The questionnaires noted the socio-demographic information of the mother-infant dyads and prelacteal feeding practices of mothers for the index infant. In this study, the dependent variable was prelacteal feeding practices. In the regression analysis, prelacteal feeding practice was coded ‘1’ while ‘0’ was coded for non-prelacteal feeding practices. The independent variables considered were: age, educational status, residence, marital status of mothers, household income, occupation, family size, sex of infant, place of delivery and ante-natal and post-natal service utilization. The age of mothers was categorized into three groups i.e. < 25, 25–35 and > 35 years. The younger age group was taken as a reference population in the regression analysis. The religion of mothers was coded as ‘0’ for Christian and ‘1’ for Muslim. Urban and rural residences of mothers were coded as ‘0’ and ‘1’ respectively. Regarding the educational status of parents, those who could not read and write were coded as ‘0’ while the rest were coded as ‘1’. Mothers who were housewives were coded ‘0’ while farmers and employed mothers were coded ‘1’. The lowest household income was coded ‘0’ while the other two levels were coded ‘1’. Mothers who received infant feeding counseling and delivered in a health institution were coded as ‘0’, while those who did not receive those services were coded as ‘1’ [21].

### Operational definitions

Prelacteal feeding: is defined as giving liquids or foods other than breast milk prior to the establishment of regular breastfeeding [6].

Early initiation of breastfeeding: are the proportion of children born in the last 24 months who were made to breastfeed within 1 h of birth [3].

Exclusive breastfeeding: are the proportion of infants less than 6 months of age, who are exclusively breastfed with breast milk and no other liquids or solids, with the exception of oral rehydration solution, supplements or medicines [3].

Health Extension Workers (HEWs): are community level health workers trained for 1 year at undergraduate level to deliver preventive, promotive and curative health services, such as maternal and child health services [24].

Health Development Army: are a network of up to 5 families each of which one of the families, who is an innovator or front liner in practicing health behavior, leads the network and gradually influences the rest of the households to acquire skills and changes in attitudes towards healthy behavior. The network is technically supported by the HEWs, who facilitate and follow up through regular conversations held within the community [24].

Kebele: is the smallest administrative unit with a population of 5000 people [25].

### Data quality control

A two-day long training and pretesting were arranged for data collectors and supervisors. Moreover, to maintain the quality of collected data, the questionnaires were pre-tested using 10% of the sample size at the Basona Worana district within North Shoa administrative zone. The data collection tools were amended based on the findings of the pilot test. Supervision was conducted during the actual data collection by the investigators. Every questionnaire was checked for completeness and supervisors were providing feedback on the quality of collected data on a daily basis. In addition, filled in questionnaires were cleaned and coded to double enter into computers by the data encoder.

### Data management and analysis procedure

Data were entered using the EPI Info statistical software V.3.5.1(CDC, Atlanta, Georgia, USA) [26] and exported to Statistical Package for Social Science (SPSS) research (SPSS-IBM- version 20) [27] for analysis. Cleaning was conducted using frequencies and univariate analysis. Percentages, frequency distributions and measures of central tendency and measures of dispersion were used for describing the data. The investigators used the ‘recall since birth’ method to determine the magnitude of prelacteal feeding practices among mother-infant dyads in the targeted community. Bivariate logistic regression was computed to identify the association of independent and dependent variables. Finally, based on the recommendations of Bendel and Afiff (1977), independent variables found to have *P*-value <=0.2 [28] were entered into multivariate logistic regressions to control the effect of confounding. The Hosmer-Lemeshow goodness-of-fit was used to test for model fitness. Results were reported as Crude Odds Ratio (COR) or Adjusted Odds Ratio (AOR) with 95% Confidence Intervals (CIs). The statistical significance test was accepted at *p* < 0.05.

### Ethical considerations

Ethical clearance was obtained from the Institute Review Board (IRB) of Debre Berhan University. Permission was granted form both Debre Berhan District Health Office and kebele administrations. Informed consents were obtained from all mothers participating in the study. Participation of all respondents in the survey was strictly voluntary. All information obtained from the respondents was anonymous and confidential.

## Results

### Socio-demographic characteristics of mother-infant dyads

In this study a total of 634 mother-infant dyads were enrolled, resulting in a response rate of 100%. The mean (±SD) age of the mothers was 30.9 years (±6.20). Slightly higher than one-fourth of mothers, 163 (25.7%) were illiterate respondents. Close to one third 208 (32.8%) of mothers were farmers. The mean age of infants with standard deviation was 7.79 months (±3.23). Slightly higher than half of the children 344 (54.3%) were male. The majority of mothers, 535 (84.4%), delivered their babies in health institutions (Table 1).

**Table 1:** Socio-Demographic characteristics of mother-infant dyads, Debre Berhan district, April 2014. Presents the socio-demographic characteristics of study participants including residence, age of mothers, religion, sex and birth order of infants. Continuous variables are described using mean, and standard deviation

Characteristics	Frequency	Percentage
Place of residence		
Urban	317	50.0
Rural	317	50.0
Age of mothers (years)		
< 25	108	17.0
25–35	353	55.7
> 35	173	27.3
Age ((Mean ± SD) Years	30.9 (±6.2)	
Religion		
Christian	574	90.5
Muslim	60	9.5
Educational status of mother respondents		
Unable to read and write	163	25.7
Read and write	180	28.4
Elementary school	105	16.6
High school & preparatory school	103	16.2
Graduate	83	13.1
Current work status of respondents		
Farmer	208	32.8
Civil servant	130	20.5
Housewife	125	19.7
Merchant	107	16.9
Daily labourer	46	7.3
Student	11	1.7
House servant (maid)	7	1.1
Monthly income of the household		
Less than 30.00 USD^¥^	217	34.2
30.00–60.00 USD	175	27.6
Greater than 60.00 USD	242	38.2
Family size (Mean ± SD) (Persons/household)	4.48 ± 1.57	
Sex of infant		
Male	344	54.3
Female	290	45.7
Age of infant (Mean ± SD) in months	7.79 ± 3.23	
Birth Order		
First	237	37.4
Second – Third	173	27.3
Forth and more	224	35.3
Age of infant (Mean ± SD) in months	7.79 ± 3.23	

### Utilization of maternal health service

Five hundred and sixty-seven (89.4%) of mothers had received at least one antenatal care service for the index infant. In addition, the majority (82.6%) of mothers were offered infant feeding counseling services (Table 2).

**Table 2:** Utilization of Maternal and Child health services in Debre Berhan district, April 2014. Presents the maternal and child health service utilization rates. The services are antenatal care, delivery, postnatal care, and infant feeding counseling services

Characteristics	Frequency	Percentage
ANC follow up		
Yes	567	89.4
No	67	10.6
ANC Visits		
1–2 visits	92	14.5
3 or more	475	85.5
Place of birth		
Health institution	535	84.4
Home	99	15.6
Received Postnatal Care		
Yes	397	62.6
No	237	37.4
Received counseling on infant feeding		
Received	524	82.6
Not received	110	17.6

### Feeding practice of mothers

Fourteen-point 2 % (95% CI: 11.00–17.00%) of mothers reported that they had provided prelacteal feed to their indexed infants. Of these, 53.3% provided butter and 40.0% provided a glucose solution made of water and sugar. Four hundred and forty-eight (70.7%) and 506 (79.8%) of mothers had initiated breastfeeding within 1 h and twenty-four hours after delivering their indexed baby, respectively. Two third of infants (68.6%) were exclusively breastfed for their first 6 months of life (Table 3).

**Table 3:** Feeding practice of mothers, Debre Berhan district, April 2014. (*n* = 634). Depicts the feeding practices of mothers for their indexed infants. Some of the characteristics presented in the table include; prelacteal feeding, time of initiation of breastfeeding, and exclusive breastfeeding practices of mothers

Characteristics	Frequency	Percentage
Pre-lacteal feed		
Yes	90	14.2
No	544	85.8
What did you give as pre-lacteal feeding		
Butter	48	53.3
Water and sugar	36	40.0
Cow’s milk	6	6.7
Time of initiation of breastfeeding		
< 1 h	448	70.7
Within the first 6 h after birth	40	6.3
Within 7–24 h	18	2.8
On the second day after birth	10	1.6
On the third day after birth	16	2.5
Don’t know	102	16.0
Colostrum feeding		
Yes	505	79.7
No	129	20.3
Weaning pattern of mothers with infants less than 6 months (*n* = 199)		
Rarely	98	49.2
Sometimes	51	25.6
Most times	27	13.6
Discontinued breastfeeding	23	11.6
Exclusive breastfeeding		
Yes	435	68.6
No	199	31.4

### Factors reported in association with early initiation of complementary feeding

In the univariate logistic regression analysis; place of residence, age of mothers, educational status of mothers, income of households, place of delivery and infant feeding counseling services were associated with prelacteal feeding practices. Multi-variable logistic regression analysis was computed and four predictor variables of prelacteal feeding practices were identified (Table 4). Home delivery was the major risk factor for practicing prelacteal feeding. Mothers who delivered their indexed infant at home had practiced prelacteal feeding over four-folds more than mothers who had delivered in a health institution (Adjusted Odds Ratio (AOR) 4.70; 95% CI: 2.56–8.60, p -value = 0.001). Mothers who did not initiate breastfeeding within an hour had practiced prelacteal feeding six-folds more than their counterpart mothers who had initiated breastfeeding within an hour (AOR 5.58; 3.21–9.46, *P*-value =0.001). Similarly, with regards to the occupation of mothers, farmers were four times more likely to practice prelacteal feedings, than their counterparts, housewives, (AOR 4.33; 95% CI: 1.73–10.81, p -value = 0.002). Mothers who can read and write were 54% less likely to practice prelacteal feeding than their counterparts, illiterate mothers, with, (AOR 0.46; 95%CI: 0.22–0.98, p- value = 0.044).

**Table 4:** Factor associated with pre-lacteal feeding practices of mothers with infants aged less than 12 months in Debre Berhan district, April 2014. Presents candidate and predictor variables of prelacteal feeding practices of mothers. The results are presented with Cruds odds ratio, Adjusted odds ratio and 95% Confidence intervals and p- values

Characteristics	Pre-lacteal feeds		Crude odds ratio (95% CI)	Adjusted odds ratio (95% CI)	*p*-value
	Yes	No			
	N (%)	N (%)			
Place of residence					
Urban	35 (38.9%)	282 (51.8%)	1	1	
Rural	55 (61.1%)	262 (48.2%)	1.69 (1.07, 2.66)^a^	0.62 (0.31, 1.25)	0.18
Religion					
Christian	86 (95.5%)	465 (85.5%)	1	1	
Muslim	4 (4.5%)	79 (14.5%)	0.40(0.14, 1.14)	0.63 (0.18, 2.16)	0.46
Age of mother (year)					
< 25	17 (18.9%)	91 (16.8%)	1	1	
25–35	52 (57.8%)	301 (55.3%)	0.92 (0.51, 1.67)	1.67 (0.77, 3.60)	0.187
> 35	21 (33.3%)	152 (27.9%)	0.74 (0.37, 1.47)	1.15 (0.48, 2.77)	0.741
Educational status of mother					
Unable to read and write	33 (30.3%)	130 (23.8%)	1	1	
Read and write	23 (31.7%)	157 (28.9%)	0.57 (0.32, 1.03)	0.46 (0.22, 0.98)	0.044
Elementary school completed	18 (15.6%)	87 (16.0%)	0.81 (0.43, 1.53)	1.53 (0.65, 3.61)	0.325
High school completed	9 (14.5%)	94 (17.3%)	0.37 (0.17, 0.82)^a^	1.51 (0.50, 4.56)	0.460
Graduate	7 (7.8%)	76 (14.0%)	0.36 (0.15, 0.86)^a^	2.14 (0.53, 8.64)	0.282
Educational status of husband					
Unable to read and write	17 (18.8%)	43 (7.9%)	1	1	
Read and write	36 (40.3%)	137 (25.2%)	0.66 (0.34, 1.30)	1.77 (0.71, 4.41)	0.218
Elementary school completed	14 (15.5%)	92 (17.0%)	0.38 (0.17, 0.85)^a^	0.85 (0.30, 2.45)	0.773
High school completed	10 (11.1%)	112 (20.6%)	0.22 (0.09, 0.53)^a^	1.00 (0.30, 3.26)	0.997
Graduate	13 (14.4%)	160 (29.4%)	0.20 (0.09, 0.45)^a^	1.14 (0.30, 4.30)	0.847
Current work status of mothers					
Housewife	12 (13.3%)	113 (20.7%)	1	1	
Working/employed	17 (18.8%)	231 (42.5%)	0.93 (0.32, 1.50)	0.62 (0.22, 1.69)	0.352
Farmer	61 (67.7%)	200 (36.7%)	2.87 (1.48, 5.56)^a^	4.33 (1.73, 10.81)	0.002
Household income					
Less than 30USD	38 (42.2%)	179 (33.06%)	1	1	
30–60 USD	29 (32.2%)	146 (26.8%)	0.93 (0.55, 1.59)	1.28 (0.65, 2.50)	0.468
Greater than 60 USD	23 (25.69%)	219 (40.2%)	0.49 (0.28, 0.86)^a^	1.19 (0.52, 2.70)	0.678
Counselled on Infant feeding					
Yes	57 (63.3%)	467 (85.8%)	1	1	
No	33 (36.6%)	77 (14.2%)	3.51 (2.14, 5.74)^a^	1.60 (0.85, 3.01)	0.143
Place of delivery					
Health Institution	50 (55.5%)	485 (89.1%)	1	1	
Home	40 (44.5%)	59 (10.9%)	6.57 (4.00, 10.79)^a^	4.70 (2.56, 8.60)	0.000
Breastfeeding initiation time					
Less or equal to 1 h	441 (76.3%)	33 (36.7%)	1	1	
Greater than 1 h	129 (23.7%)	57 (63.3%)	5.57 (3.46, 8.90)^a^	5.58 (3.21, 9.69)	0.000

^a^Statistically significant variables at *p* < 0.05, *CI* Confidence Interval. Hosmer-Lemeshow goodness-of-fit = 0.273

## Discussion

This study found statistically significant association between home delivery, initiation of breast feeding within an hour, occupation and educational status of mothers and prelacteal feeding practices. The identified predictors of prelacteal feeding practices are in line with global and national identified gaps to address through this study, proper and tailored interventions are required so that the district health systems [5, 16].

The study revealed that one-sixth of mothers practice prelacteal feeding. This prelacteal feeding practice prevalence was found in line with the reported prelacteal feeding practice rates Bahir Dar city within the Amhara region at 15%, [13], and 16.8% prevalence rate reported in the Dubti town of the Afar regional state. However, the findings were much lower than the national estimate at 28.9% [7], and another reports indicating a prevalence rate of 45.4% in the Hareri region [8]. These variations could be due to differences in the study periods, study design and study participants. Furthermore, this study utilized study participants in central Ethiopia, managed in town administrations, whereas the control studies stated above were analyzed data, collected across whole regions from 576 clusters [7]. Another factor for the differences in findings is that the sampled communities reside in a rural setup with major cultural differences between one another [8, 10, 11].

In the study area, mothers reported to administering three common prelacteal feeds to their infants, namely, raw butter (53.3%), sugar and water solution (40.0%) and diluted cow’s milk (6.7%). This finding was in line with findings in different parts of the country [10, 12].

Mothers who can read and write were 54% less likely to practice prelacteal feeding than their counterpart illiterate mothers. Mothers’ optimum infant feeding practices has a significant positive effect on their behavior during postnatal periods. This finding was in line with Tamiru et al. (2013) who documented that in Jimma Arjo district [29], mothers who were exposed to breastfeeding education practiced optimal child feeding practices. In contrast, the findings contradicted Biks et al. (2015) where the education of a mother did not show statistical significance in relation to optimum breastfeeding practices [30].

The occupation of mothers showed an impact on prelacteal feeding practices. In this study, farmer mothers were up to four folds more likely to practice prelacteal feedings than their counterparts, housewives. This finding was not consistent with a study conducted in the Afar region Liben et al. 2017 [11] and Liben et al. 2016 [31] where housewives were more likely to feed their infant prelacteal feeds. This could be due to differences in study subjects, since in this study, urban and rural residents were enrolled while the other studies were conducted in rural communities.

Another finding indicated that mothers who did not initiate breastfeeding within an hour were more likely to practice prelacteal feeding. This finding was consistent with a study conducted in the Afar region where mothers who initiated breastfeeding after 1 h of delivery were nearly three times more likely to practice prelacteal feeding when compared with women who initiated breastfeeding within an hour [11]. This could be due to the fact that mothers who get the support of skilled birth attendants in health institutions may also be assisted with early initiation of breastfeeding.

Utilization of maternal, neonatal and child health services such as antenatal care, infant feeding counseling and postnatal services were positively associated with the reduction of prelacteal feeding practices in the study area. Mothers who delivered their indexed infants at home had a fourfold increased chance of engaging in prelacteal feeding practices when compared with mothers who delivered in health institutions. This finding was consistent with Legesse et al. (2014) and Bekele et al. (2014) who reported that mothers who delivered at home were from four to seven times more likely to practice prelacteal feedings [8, 10].

## Conclusions

One-sixth of mothers still practice prelacteal feeding. Ability to read and write, utilization of institutional delivery and receiving counseling services on infant feeding were the predictors for practicing prelacteal feeding. Therefore, improving access to formal and informal education for mothers, improving access to information on newborn feeding practices and encouraging mothers to deliver their infants in health institutions are recommended.

## Abbreviations

AOR: Adjusted Odds Ratio
CI: Confidence Interval
COR: Crude Odds Ratio
HSTP: Health Sector Transformation Plan
IYCF: Infant and Young Child Feeding
MDGs: Millennium Development Goals
SD: Standard Deviation
SDGs: Sustainable Development Goals
WHO: World Health Organization

## Authors’ information

MDA: MPH, Senior Health Systems Strengthening Adviser at USAID Transform: Primary Health Care, JSI Research & Training Institute Inc., Addis Ababa, Ethiopia.

MMA: MPH, Urban Health Extension Program Focal at Lalibela Town Health Office, Ethiopia.

MBA: MSC, Lecturer at Woldia University, Ethiopia.

BFD: MPH, Deputy Chief of Party at USAID Transform: Primary Health Care, JSI Research &Training Institute, Inc., Addis Ababa, Ethiopia.

TRM: DCur; Professor (full) and Executive Editor: Africa Journal of Nursing and Midwifery, University of South Africa, Department of Health Studies, Pretoria, South Africa.

KDG: Ph.D. in Health Studies; Associate Professor at Wolaita Sodo University, School of Public Health, College of Health Sciences and Medicine, Wolaita Sodo, Ethiopia.

AHF: MPH, Quality Improvement Adviser at USAID Transform: Primary Health Care, Pathfinder International, Addis Ababa, Ethiopia.

ADM: MPH, Immunization and Surveillance Adviser, USAID Transform: Primary Health Care, JSI Research & Training Institute Inc., Addis Ababa, Ethiopia.

AYD: MPH, Monitoring & Evaluation Officer at Clinton Health Access Initiative, Addis Ababa Ethiopia.
